# Monolithic full-color active-matrix micro-LED micro-display using InGaN/AlGaInP heterogeneous integration

**DOI:** 10.1038/s41377-023-01298-w

**Published:** 2023-10-30

**Authors:** Longheng Qi, Peian Li, Xu Zhang, Ka Ming Wong, Kei May Lau

**Affiliations:** Department of Electronic and Computer Engineering, The Hong Kong University of Science and Technology, Clear Water Bay, Kowloon, Hong Kong China

**Keywords:** Inorganic LEDs, Displays

## Abstract

A prototype of full-color active-matrix micro-light-emitting diode (micro-LED) micro-display with a pixel density of 391 pixel per inch (ppi) using InGaN/AlGaInP heterogeneous integration is demonstrated. InGaN blue/green dual-color micro-LED arrays realized on a single metal organic chemical vapor deposition (MOCVD)-grown GaN-on-Si epiwafer and AlGaInP red micro-LED arrays are both monolithically fabricated, followed by the integration with a common complementary metal oxide semiconductor (CMOS) backplane via flip-chip bonding technology to form a double-layer thin-film display structure. Full-color images with decent color gamut and brightness are successfully displayed through the fine adjustment of driving current densities of RGB subpixels. This full-color display combines the advantages of high quantum efficiency of InGaN material on blue/green light and AlGaInP material on red light through heterogeneous integration and high pixel density through monolithic fabrication approach, demonstrating the feasibility and prospects of high brightness, good color performance, and high-resolution micro-LED micro-displays in future metaverse applications.

## Abstract (web summary)

We develop a double-layer thin-film structure for active-matrix displays using InGaN/AlGaInP heterogeneous integration and demonstrate a prototype of full-color micro-LED micro-display with a pixel density of 391 ppi.

## Graphical abstract

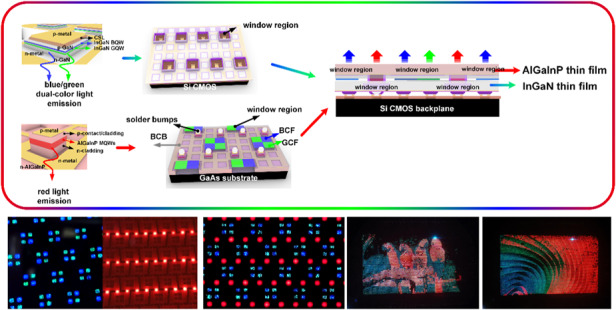

## Introduction

Micro-LEDs are regarded as a promising display technology for micro-display applications such as augmented/mixed reality (AR/MR), which require high brightness and high resolution while featuring a small display size^1–4^. However, the development of micro-LED display technology is accompanied by difficulties^5,6^, in which realizing acceptable full-color emission is one of the challenging issues to be resolved for practical applications of micro-LED displays.

For high-resolution full-color micro-LED displays, mass transfer technology has not been adopted in general due to its limitation in pixel pitch and transfer alignment accuracy^7^. The color down-conversion method is a preferred choice and has been extensively studied over the past few years. By using monolithically fabricated micro-LED arrays emitting either UV^8^ or blue light^9^ to optically pump the color-converting materials such as quantum dots (QDs) or nanophosphors, full-color micro-LED displays have been demonstrated from pixel array level^10–12^ to panel level^13,14^. The monolithically fabricated micro-LED array is suitable for the realization of micro-displays because of its chip-level fabrication, making it straightforward to implement a high pixel density. The monolithic micro-LED display chip can be further realized with the integration of CMOS backplanes using flip-chip bonding^15,16^ or direct wafer-level bonding^17,18^. Despite of these advantages, the brightness and color performance of color-converted full-color micro-LED displays are highly dependent on the conversion and absorption efficiency of the color-converting materials, which also need to be further improved to meet the requirements of high brightness and wide color gamut for practical applications^19^.

In addition to the color down-conversion approach, heterogeneous integrations of InGaN and AlGaInP LEDs have been reported. Full-color LED pixels were achieved by adhesive bonding of InGaN blue and green LED or a dual-color LED with an AlGaInP LED^20,21^, without the integration to display drivers. The InGaN dual-color LED, consisting of separate blue and green active regions, was grown on sapphire using selective area and regrowth approach. But to make a display, the effective regrowth of LED epiwafers and addressing of all RGB LED subpixels need to be further explored. Tandem full-color LEDs were proposed using epitaxy laser lift-off^22^ or 2D materials-based transfer^23^, followed by a transfer printing process to integrate large-size InGaN blue, green and AlGaInP or AlGaAs red LEDs in a vertical stacking structure. Theoretically, higher resolution can be achieved by applying such vertical integration rather than a horizontal one. Still, complex addressing of all the vertically stacked RGB LED subpixels is necessary when processing it into a high-resolution display. A heterogeneously integrated full-color display was demonstrated by direct bonding of InGaN blue, green, and AlGaInP red in three different processed LED epiwafers^24,25^. The bonded epiwafers with vertically stacked RGB LEDs were further integrated with the CMOS backplane. Nevertheless, the process is extremely complex involving multiple times of wafer-bonding. The fabrication yield is of serious concern at each stage of wafer bonding. Previously, we successfully demonstrated a low-resolution full-color passive-matrix micro-LED display by heterogeneous integration of GaN-on-sapphire and AlGaInP LED arrays^26^. To fully exploit the high quantum efficiency of InGaN and AlGaInP material in blue/green and red light wavelength region, respectively, developing high-resolution and active-matrix addressable micro-LED displays with a simplified fabrication process by such heterogeneous integration approaches is remarkably attractive.

Here, we develop a double-layer thin-film structure for active-matrix displays using the heterogeneous integration of GaN-on-Si and AlGaInP material systems. A prototype of full-color micro-LED micro-display with a resolution of 200 × 80 and a pixel density of 391 ppi is further demonstrated. The blue/green dual-color emissions are obtained from our self-grown GaN-on-Si LED epiwafers containing a single active region, and red subpixels are made using commercial AlGaInP LED epiwafers. The InGaN blue/green dual-color micro-LEDs arrays as well as AlGaInP red micro-LED arrays are both monolithically fabricated and sequentially integrated with a common CMOS backplane via Au-Sn and Au-In flip-chip bonding, respectively. The heterogeneous integration of two monolithic arrays leads to full-color displays with a wide color gamut, high brightness, and high pixel density. This approach provides an alternative feasible solution for achieving high-resolution full-color active-matrix micro-LED micro-displays with simplified fabrication beyond the color conversion method and multi-stack wafer bonding technology.

## Results

**Fig. 1:**
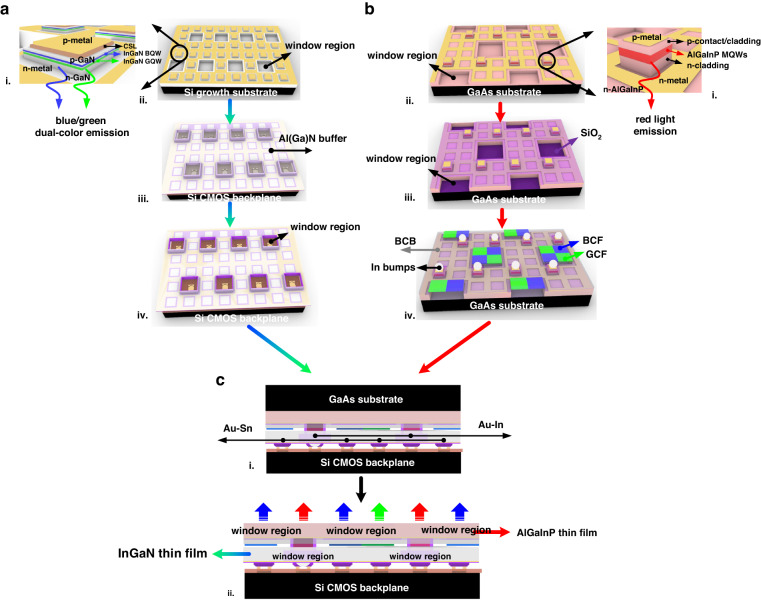
Process flow of the double-layer thin-film full-color micro-LED micro-display by heterogeneous integration. **a** Fabrication of InGaN blue/green dual-color micro-LED display: i. schematic of InGaN dual-color micro-LED subpixel; ii. after window regions definition and p, n metallization; iii. after Au-Sn flip-chip bonding of InGaN dual-color micro-LED array and CMOS backplane; iv. after the Al(Ga)N buffer etchback. **b** Fabrication of AlGaInP red micro-LED array: i. schematic of AlGaInP red micro-LED subpixel; ii. after window regions definition and p, n metallization; iii. after SiO_2_ passivation and contact holes opening; iv. after BCB etchback, reflow of In micro bumps and blue/green color filter patterning. **c** Heterogeneous integration: i. Au-In flip-chip bonding of InGaN dual-color display and AlGaInP red micro-LED array; ii. GaAs substrate removal

**Fig. 2:**
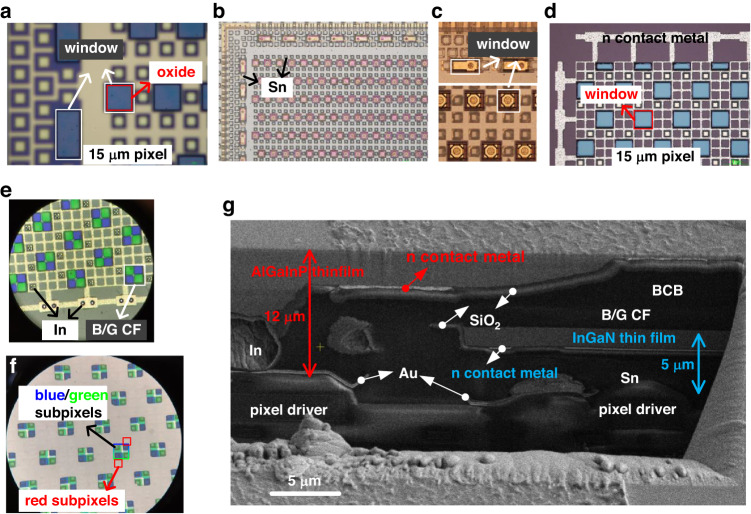
Process inspections of heterogeneously integrated full-color micro-LED display. **a** SiO_2_ passivation of InGaN dual-color micro-LED array. **b** InGaN blue/green dual-color micro-LED display with Si growth substrate removal. **c** Al(Ga)N buffer etchback to open the window regions on the InGaN thin film. **d** AlGaInP red micro-LED array with metalization and window regions. **e** AlGaInP red micro-LED array with In micro bumps and blue/green color filter patterning. **f** Full-color display after GaAs substrate removal. **g** SEM image of the double-layer thin-film structure

The heterogeneous integration of the monolithically fabricated InGaN blue/green dual-color micro-LED display and AlGaInP red micro-LED arrays is achieved by flip-chip bonding technology. Figure 1 shows the layout and the fabrication process flow of the double-layer thin-film full-color micro-LED display. Different from traditional monochromatic LEDs, the relative emission intensity of blue and green light in dual-wavelength epiwafers can be adjusted by applying different current densities or employing different configurations of blue and green quantum wells (QWs). Research of blue/green dual-color LED devices on the sapphire substrate^27–29^ has been reported in the past. Inspired by those results, we grew 4-inch dual-wavelength GaN-on-Si LED epi-wafers by stacking three pairs of bottom blue QWs, one pair of green QW, and two pairs of top blue QWs as the active region. The fabrication process of the blue/green dual-color GaN-on-Si micro-LED display is similar to our previous report of high-resolution and high-brightness GaN-on-Si blue monochromatic display^30^. Notably, window regions with a size of 30 μm × 30 μm are specially designed for the heterogeneous integration of the AlGaInP micro-LED arrays. The dual-color subpixels are then defined and integrated with a CMOS backplane, followed by the Si growth substrate removal. An additional etch-back process was performed to etch through the window regions (Fig. 1a). Figure 2a presents the microscope image of the InGaN micro-LED array after SiO_2_ passivation. The size of oxide openings is smaller than the window regions to prevent short-circuit of red subpixels during the flip-chip bonding of the AlGaInP red micro-LED array. The microscope images of blue/green dual-color display before and after Al(Ga)N buffer etch-back are shown in Fig. 2b and 2c, respectively, where window regions for the bonding of both p- and n-electrode are presented.

Fabrication of the red micro-LED arrays was performed using commercially available 4-inch AlGaInP red LED epiwafers grown on GaAs substrate, which is similar to the fabrication of GaN-on-Si dual-color micro-LED arrays (Fig. 1b). To allow blue/green light from the bottom InGaN active layer to transmit through the AlGaInP active layer, 50 μm × 50 μm window regions were etched accordingly. The process inspections of the as-fabricated monolithic AlGaInP red micro-LED arrays are shown in Fig. 2d and 2e. The AlGaInP red micro-LED array was then flip-chip bonded to the as-prepared blue/green dual-color micro-LED display through the window regions on the InGaN thin film, followed by the GaAs substrate removal (Fig. 2f). An epoxy underfill process is necessary for the bonding of AlGaInP red micro-LED array, which effectively supports the fragile AlGaInP thin film and improves its mechanical strength. The reliability of the double-layer heterogeneous structure can then be further enhanced by curing the epoxy to obtain a crack-free and firm AlGaInP surface. In this double-layer thin-film display, blue and green light are transmitted through the window regions on AlGaInP thin film and red light emits from the AlGaInP active layer at the positions of window regions on the InGaN thin film. The SEM image demonstrates the cross-section of this heterogeneous full-color display (Fig. 2g).

**Fig. 3:**
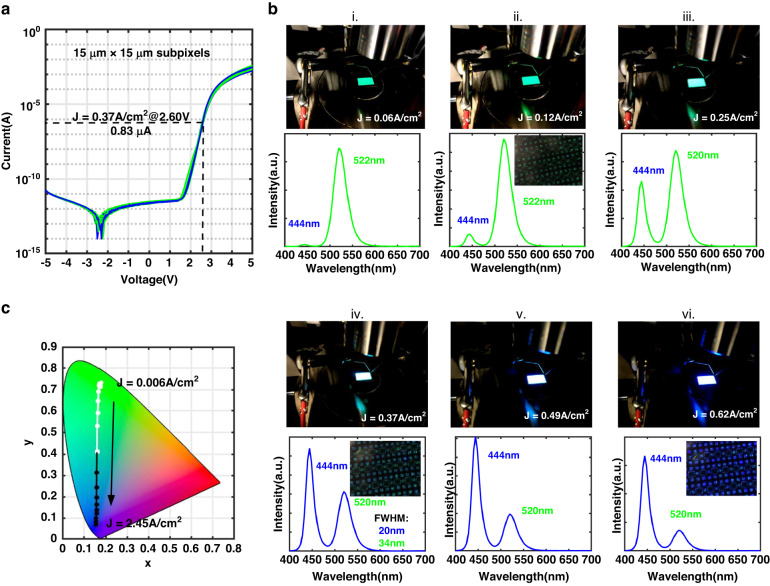
Characterization of InGaN blue/green dual-color micro-LED diaplay. **a** *I*–*V* curves of InGaN dual-color micro-LEDs. **b** EL spectra and corresponding photos of InGaN blue/green dual-color display at different current densities. **c** Color coordinates of InGaN blue/green dual-color display at different current densities in CIE 1931 color space

Current-voltage (*I–V*) characteristics of the InGaN dual-color micro-LED subpixels were measured after the metallization step (Fig. 3a). The reverse leakage current at −5 V is 19.5 pA. To investigate the behaviors of blue/green dual-color emissions, all subpixels of the InGaN dual-color display were turned on by directly injecting DC current. Figure 3b presents the electroluminescence (EL) spectra of the dual-color display in which the intensity and ratio of dual-wavelength peaks vary with current densities. The InGaN dual-color display produces a pure green color when the current density is lower than 0.12 A/cm^2^ (Fig. 3b-i, ii). Continuing to increase the current density to 0.25 A/cm^2^, the blue peak rapidly increases and becomes almost comparable to the green peak (Fig. 3b-iii). When the current density is raised to 0.37 A/cm^2^, a turning point of dual-color emissions occurs and the blue peak becomes the dominant one while the ratio of blue-to-green peak is still comparable. It clearly shows that each InGaN micro-LED subpixel simultaneously emits proportional green and blue light, displaying appropriate dual-color emissions (Fig. 3b-iv). Corresponding full width at half maximum (FWHM) of the EL spectrum for blue and green peaks are 20 nm and 33 nm. Further increasing the current density to 0.62 A/cm^2^, both blue and green peaks grow together and the blue peak becomes the decidedly dominant peak (Fig. 3b-v, vi). The peak wavelengths of blue/green dual-wavelength emission, which are around 444 nm and 520 nm, respectively, stay almost unchanged within the measured current range. Figure 3c displays the shift of color coordinates in CIE 1931 chromaticity diagram of InGaN blue/green dual-color display when the current density is increasing. To provide a comparable intensity of blue and green light, we set the operating current density of each dual-color InGaN micro-LED at 0.37 A/cm^2^, corresponding to the current of 0.83 µA and voltage of 2.60 V.

From the measurements, we observe that only green QW in the active region emits light under ultra small injection conditions. This is because that the limited injecting carriers are primarily distributed in the green QW because of the quasi-fermi levels reach the band of green QW first. With the increase of carrier injection, more carriers fill and recombine both in the top blue and green QWs so that the blue peak gradually occurs and increases together with the green peak. However, the ratio of blue-to-green emission keeps enlarging with the increase of current density. This is plausibly attributed to the fact that the carriers overflow from the green QW to the top blue QWs, and meanwhile holes density starts to increase in the bottom blue QWs under high current densities. Consequently, the carriers recombination rate in the blue InGaN QWs exceeds green InGaN QW, resulting in a stronger and faster-growing blue peak.

**Fig. 4:**
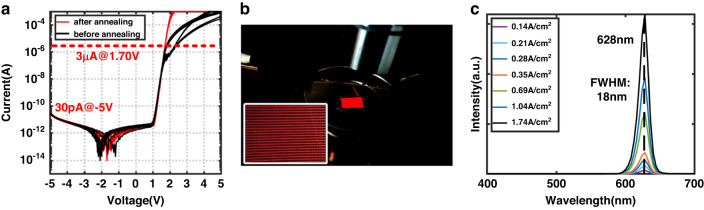
Characterization of AlGaInP red micro-LED array. **a** *I*–*V* curves of AlGaInP red micro-LEDs. **b** Photos of AlGaInP red micro-LED array after Au-In bonding. **c** EL spectra of AlGaInP red micro-LEDs at different current densities

The *I–V* characteristics of AlGaInP red subpixels are shown in Fig. 4a. Before p and n contact metal annealing, the *I–V* curves of micro-LEDs in the array are non-uniform. Large series contact resistance can be observed at the forward-biased region. After annealing, the contact resistance dramatically decreased and uniform subpixels *I–V* curves were obtained. Total series resistance extracted from *I–V* curves (<1 mA) was decreased from 1.2 kΩ to 230 Ω for red micro-LEDs. The reverse leakage current of red micro-LEDs at −5 V is around 30 pA. The forward voltage of red micro-LEDs is 1.70 V at 3 µA. Uniform emission and high yield were observed when all the red subpixels were turned on (Fig. 4b). The yield of red subpixels is highly associated with the Au-In bonding quality and underfilling process after the bonding of AlGaInP micro-LED arrays. The small gap between AlGaInP thin film layer and GaN thin film layer will cause difficulties in epoxy underfilling and result a low display yield. The peak wavelength in red light EL spectra was measured as 628 nm within the current density range from 0.14 A/cm^2^ to 1.74 A/cm^2^, and FWHM is around 18 nm (Fig. 4c).

**Fig. 5:**
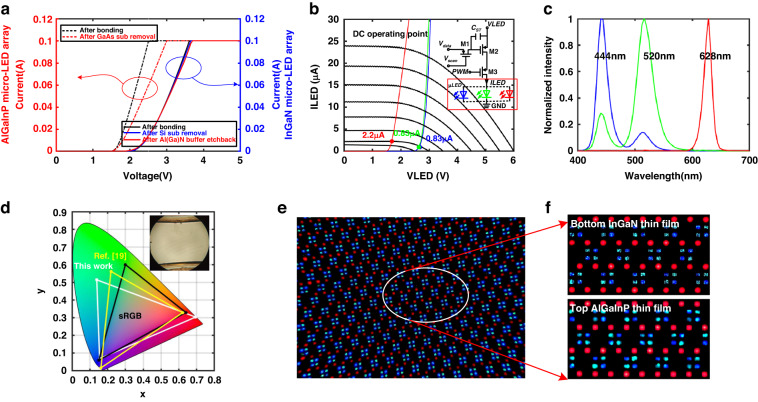
Characterization of heterogeneously integrated full-color micro-LED display. **a** *I*–*V* curves of InGaN dual-color micro-LED array and AlGaInP red micro-LED array during the integration process. **b** Pixel driver output curves and *I*–*V* curves of InGaN blue/green dual-color and AlGaInP micro-LED subpixels. **c** EL spectrum of the full-color display (VLED = 3.2 V). **d** Color gamut in CIE 1931 chromaticity diagram (Inset is the as-fabricated double-layer thin-film full-color display). **e** Inspection of all RGB subpixels turned on. **f** Zoomed-in photos at different focal planes

The operating behaviors of InGaN blue/green dual-color subpixels and AlGaInP red subpixels were analyzed after the heterogeneous integration. We measured the *I–V* characteristics of the whole InGaN dual-color micro-LED array and AlGaInP micro-LED array at each integration step. As shown in Fig. 5a, the *I–V* curves of the InGaN dual-color micro-LED array remained unaffected after flip-chip bonding, Si growth substrate removal and Al(Ga)N buffer etch-back process. But the electrical performance of the AlGaInP red micro-LED array was degraded after GaAs substrate removal, possibly because the current conduction path in the array is limited in the several microns thick n-AlGaInP layer after removal of the thick conductive GaAs substrate. Owing to this resistance degradation, the *I–V* curve of a single red subpixel was modified and plotted together with the *I–V* curves InGaN blue/green dual-color subpixels in Fig. 5b. Digitally driven three-transistor-one-capacitor (3T1C) pixel drivers are used to drive the RGB subpixels. Grayscale is provided by modulating the pulse width of the *V*_*data*_ signal. Only on and off states exist in the pixel driver, corresponding to the *V*_*data*_ signal set at *V*_*low*_ and *V*_*high*_, respectively. The on-state current is determined by the intersection point of pixel output curves and *I–V* curves of InGaN dual-color and AlGaInP red subpixels. As we discussed above, the InGaN blue/green dual-color micro-LEDs were chose to be driven at 0.83 µA thus to provide comparable intensity of blue and green light. The on-state DC current of red subpixel is then determined to be around 2.2 µA because the RGB subpixels share the same pixel driver layout. Considering the bonding resistance and possible pixel level nonuniformity, we set the VLED at 3.2 V.

The EL spectra of the full-color display with RGB monochromatic emissions are shown in Fig. 5c. The blue and green emission from the InGaN dual-color active layer using blue and green color filters are not as pure as the red light (Supplementary Fig. S4). The peak wavelengths of the blue, green, and red light are 444 nm, 520 nm, and 628 nm. Their (*x*, *y*) coordinates in CIE 1931 color space are (0.154, 0.069), (0.138, 0.515), and (0.695, 0.304) respectively. Compared with our previously reported work using the QDs-photoresist color conversion method^19^, this heterogeneously integrated full-color display presents better red emission, with less ideal blue and green color performance (Fig. 5d). The color gamut is estimated to be 109% of sRGB and brightness is around 300 nits at the aforementioned low operating current densities. In fact, a much higher level of brightness can be achieved by directly increasing the driving current of pixel drivers via VLED. The display brightness can reach over 4000 nits when the operating current densities are 2.5 A/cm^2^ and 3.4 A/cm^2^ for InGaN blue/green dual-color subpixels and AlGaInP red subpixels, respectively. But in this case the color mixing will be a trouble since the intensity of green light is much weaker than blue light, which is limited by the GaN-on-Si dual-wavelength LED epiwafers we grew.

**Fig. 6:**
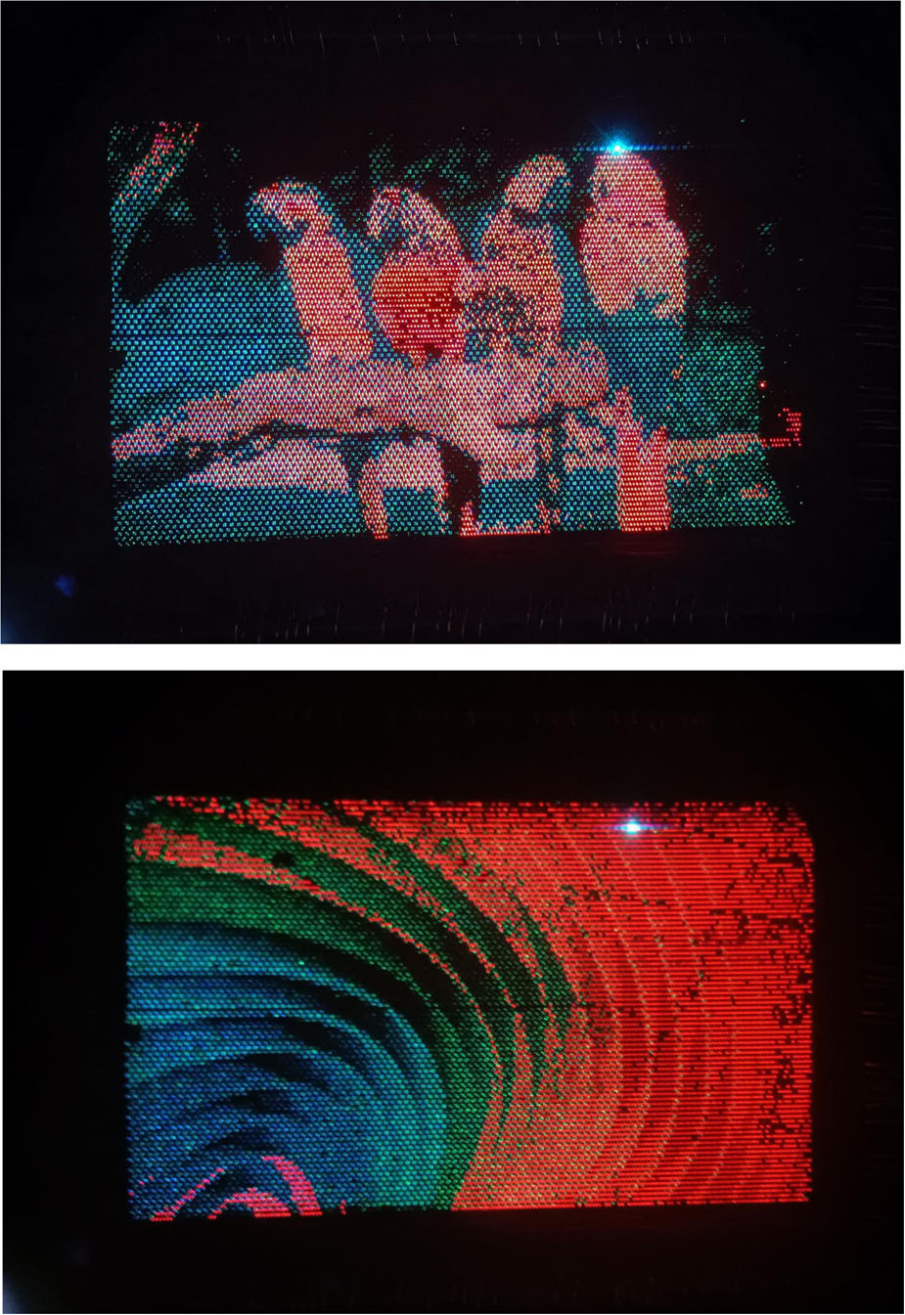
Demonstration of full-color images (0.55 in. in diagonal)

Distinguishable RGB subpixels are observed from the microscope images when all subpixels are turned on (Fig. 5e). A single full-color pixel consists of three RGB subpixels. To achieve a better current spreading effect for the common n-AlGaInP layer and compromise the layout constraints of the CMOS backplane, six pixel drivers are occupied by a single full-color pixel, which features a size of 60 µm × 90 µm (Supplementary Fig. S6). The blue/green light emitting plane and red light emitting plane are not confocal because of the height difference of active layers in the heterogeneous integration structure. The zoomed-in images focusing on the emission plane of the InGaN thin film and AlGaInP thin film are shown in Fig. 5f. A stronger red emission was expected compared with the blue and green emissions when directly giving DC current without addressing. This is because red micro-LED subpixels have lower forward voltages than the blue/green subpixels so the driving current for the red is much easier to be saturated, as we discussed in Fig. 5b. By setting VLED at 3.2 V and properly tuning the grayscales of RGB subpixels (R:G:B = 1:2:5), full-color images and video with the best color mixing effect were demonstrated (Fig. 6, Supplementary Fig. S8, Movie S1). The yield of this heterogeneous full-color display is primarily determined by the integration step of the monolithic AlGaInP red micro-LED array.

## Discussion

By using the heterogeneous integration of InGaN and AlGaInP material systems, we develop a double-layer thin-film display structure for full-color active-matrix micro-LED micro-displays. A prototype of full-color display with a resolution of 200 × 80 is successfully demonstrated. The performance of the current prototype is limited by the yet-to-be-optimized GaN-on-Si dual-wavelength LED epiwafers and the CMOS driver. To achieve high brightness with a good color mixing effect, the active region growth of dual-wavelength LED epiwafers needs to be further optimized thereby pushing the operating current density of InGaN dual-color subpixels featured comparable blue and green emission to a higher level. A possible method is to increase the number of green QWs to intensify the green emission at low current density. At the same time, the pixel driver for RGB subpixels requires individual design so that the on-state DC current of AlGaInP red subpixel and InGaN blue/green subpixels can be independently adjusted to realize a better color performance. Much higher brightness can be expected by increasing the operating current densities of RGB subpixels with the optimization of the growth of GaN-on-Si dual-wavelength LED epiwafers and the design of pixel drivers. For small-size micro-display applications such as AR/MR, we expect this monolithic and heterogeneous integration approach will be a promising option in the future.

## Materials and methods

The dual-wavelength InGaN LED epiwafers were grown on 4-inch Si substrates in our metal-organic chemical vapor deposition system. Starting with a 1.4-µm-thick Al(Ga)N buffer layer, 1.5 µm n-GaN was grown on it, followed by the dual-wavelength InGaN/GaN MQWs active region, a 50 nm AlGaN/InGaN electron blocking layer and a 250 nm p-GaN layer on top. The InGaN/GaN MQWs region is composed of three pairs of blue QWs, one pair of green QW and two pairs of blue QWs from bottom to top (Supplementary Figs. S1, S2). The mesa of dual-color subpixels with a size of 15 μm × 15 μm was patterned using an ITO self-alignment process by the Cl_2_-based inductively coupled plasma (ICP) etching. Cr/Al-based metal stack was deposited on ITO and n-GaN as p and n contact metal, respectively (Fig. 1a-ii). A 300-nm-thick SiO_2_ layer and a transparent overcoat photoresist were subsequently used to passivate and planarize the dual-color micro-LED arrays. Contact holes were opened on the p and n contact pad regions to facilitate the reflow of tin (Sn) solder bumps (Supplementary Fig. S3). Finally, the GaN-on-Si blue/green dual-color micro-LED array was flip-chip bonded onto a CMOS backplane via Au-Sn bonding, followed by Si growth substrate removal, to allow the reflected blue/green light to emit upwards. A 500 nm thin Al(Ga)N buffer layer was intentionally kept during the etching process of window regions, acting as an etch stop layer during the Si substrate removal (Fig. 1a-iii). To etch through the window regions, an etch-back process was performed (Fig. 1a-iv).

The 4-inch AlGaInP epiwafers consist of a thick n-type AlGaInP layer and p-type GaP contact layer, as well as the AlGaInP MQWs and cladding layers (Fig. 1b-i). The total thickness of all the epilayers is around 8 μm. The mesa structure was self-aligned to the p contact metal pad of each red subpixel and etched down together with the remaining epilayers in the window regions. The size and height of each mesa are 15 μm × 15 μm and 4 μm, respectively. Selective wet etching was performed to remove the un-etched materials in the window regions until smooth and mirror-like GaAs substrate was exposed. The undercut of window regions must be well controlled during the wet etching process to prevent possible failure of neighboring mesas and n metal lines. Then p contact metal was annealed in N_2_ ambiance to form a good ohmic contact with the p-GaP layer. Similarly, a metal stack layer consisting of Ge/Au was deposited as n contact metal and treated with annealing (Fig. 1b-ii). A layer of 1-μm-thick SiO_2_ was deposited to passivate the mesa sidewalls. Contact holes on the p and n contact metal pads were opened to assist solder metal reflow (Fig. 1b-iii). Thick oxide also acts as an etch stop layer in the window regions during the GaAs substrate removal process. Afterwards, 13-μm-thick BCB was over-coated to planarize the surface morphology and cured to increase the mechanical stability of the red micro-LED arrays. A BCB etch-back process was then individually performed to expose both p and n contact holes. Blue and green color filters were patterned at the positions of window regions to selectively filter the blue and green light from InGaN active layer. Finally, indium (In) pads were deposited on the p and n contacts holes of red subpixels (Supplementary Fig. S5) and reflowed into micro solder bumps, which are around 10 μm and 7 μm, respectively (Fig. 1b-iv).

The heterogeneously integrated system has two different types of bonding structure, in which the bottom InGaN blue/green dual-color side adopts Au-Sn bonding and the upper AlGaInP red side adopts Au-In bonding (Fig.1c-i). The Au-In bonding of the red micro-LED array has a lower soldering temperature (180 °C) than the Au-Sn bonding (220 °C) so that it will not damage the as-fabricated InGaN dual-color display structures. With proper underfill and the protection of silicone, the opaque GaAs substrate was further removed by an ammonia-based mixed solution^31^, forming a double-layer thin-film structure (Fig.1c-ii, Supplementary Fig. S7).

### Supplementary information

supplementary information

supplementary information Movie S1
