# Supplementary material for: Monolithic full-color active-matrix micro-LED micro-display using InGaN/AlGaInP heterogeneous integration
Source: Light Sci Appl. 2023 Oct 30;12:258. doi: 10.1038/s41377-023-01298-w (PMC10613616; doi:10.1038/s41377-023-01298-w)
Supplement: Supplementary file 1 — supplementary information [file 41377_2023_1298_MOESM1_ESM.pdf]

Supplementary Information for

Monolithic full-color active-matrix micro-LED micro-display using InGaN/AlGaInP heterogeneous integration

Longheng Qi, Peian Li, Xu. Zhang, Ka Ming Wong and Kei May Lau*

Department of Electronic and Computer Engineering, The Hong Kong University of Science and Technology, Clear Water Bay, Kowloon, Hong Kong, China.
*Email: eekmlau@ust.hk

This PDF file includes:

MATLAB Codes for images demonstration;
Figures S1 to S8;
Legends for Movies S1;

Other supporting materials for this manuscript include the following:

Movies S1

MATLAB codes for image demonstration

```
%% Random full-color image with a resolution 200×80 displayed on a 400×240 panel
RGB = imread('IMAGE.jpg'); % IMAGE.jpg is a 200×80 full-color image
r = RGB(:,:,1); % extract red color value
g = RGB(:,:,2); % extract green color value
b = RGB(:,:,3); % extract blue color value
Bayer = zeros(240,400); % initialize a Bayer matrix to store RGB value
for i = 1:1:40
    for j = 1:1:100
        % pixel A
        Bayer(3*(2*i-1)-2,2*(2*j-1)-1) = 2*g(2*i-1,2*j-1);
        Bayer(3*(2*i-1)-2,2*(2*j-1)) = 5*b(2*i-1,2*j-1);
        Bayer(3*(2*i-1)-1,2*(2*j-1)-1) = 1*r(2*i-1,2*j-1);
        Bayer(3*(2*i-1)-1,2*(2*j-1)) = 0;
        Bayer(3*(2*i-1),2*(2*j-1)-1) = 0;
        Bayer(3*(2*i-1),2*(2*j-1)) = 0;

        % pixel B
        Bayer(3*(2*i-1)-2,2*(2*j)-1) = 0;
        Bayer(3*(2*i-1)-2,2*(2*j)) = 0;
        Bayer(3*(2*i-1)-1,2*(2*j)-1) = 1*r(2*i-1,2*j);
        Bayer(3*(2*i-1)-1,2*(2*j)) = 0;
        Bayer(3*(2*i-1),2*(2*j)-1) = 2*g(2*i-1,2*j);
        Bayer(3*(2*i-1),2*(2*j)) = 5*b(2*i-1,2*j);

        % pixel C
        Bayer(3*(2*i)-2,2*(2*j-1)-1) = 0;
        Bayer(3*(2*i)-2,2*(2*j-1)) = 0;
        Bayer(3*(2*i)-1,2*(2*j-1)-1) = 0;
        Bayer(3*(2*i)-1,2*(2*j-1)) = 1*r(2*i,2*j-1);
        Bayer(3*(2*i),2*(2*j-1)-1) = 5*b(2*i,2*j-1);
        Bayer(3*(2*i),2*(2*j-1)) = 2*g(2*i,2*j-1);

        % pixel D
        Bayer(3*(2*i)-2,2*(2*j)-1) = 5*b(2*i,2*j);
        Bayer(3*(2*i)-2,2*(2*j)) = 2*g(2*i,2*j);
        Bayer(3*(2*i)-1,2*(2*j)-1) = 0;
        Bayer(3*(2*i)-1,2*(2*j)) = 1*r(2*i,2*j);
```

```
        Bayer(3*(2*i),2*(2*j)-1) = 0;
        Bayer(3*(2*i),2*(2*j)) = 0;
    end
end
Y = uint8(Bayer); % convert data storage type 'Bayer(double)' to the image data
type 'Bayer(uint8)'
imshow(Y)% show grayscale image
imwrite(Y,'IMAGE.png'); % save image in current folder
```

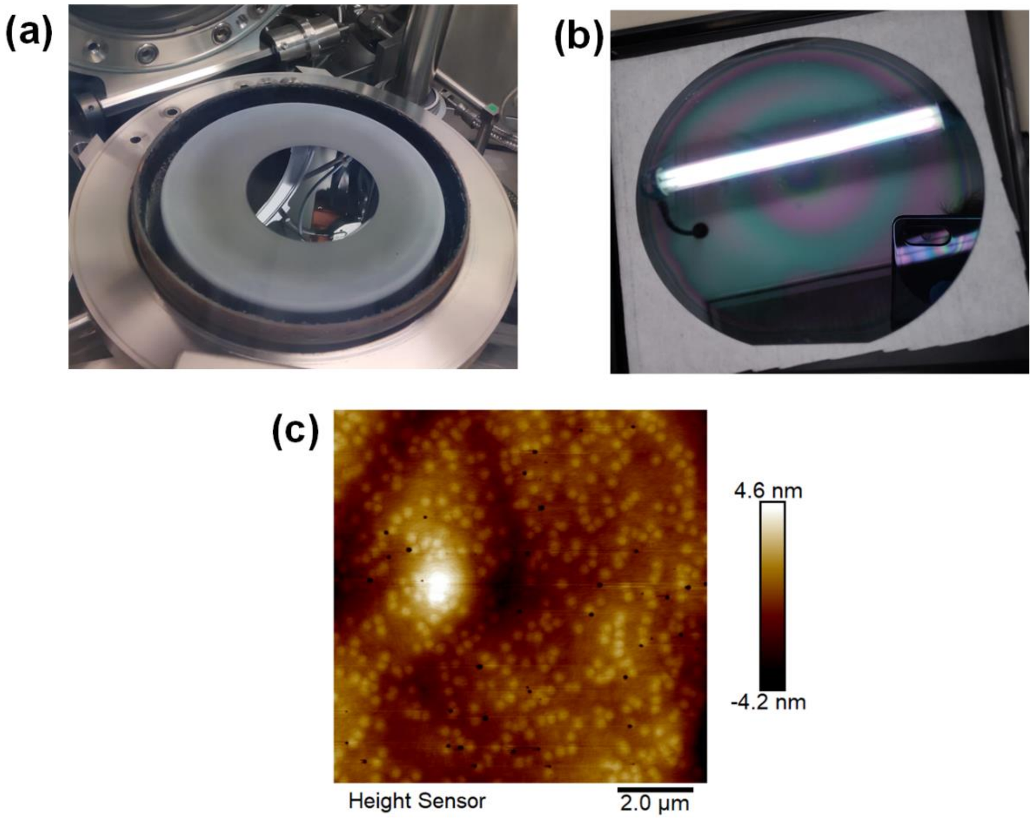

Figure S1. Growth of 4-inch GaN-on-Si dual-wavelength epiwafers. (a) 4-inch sample grown in our MOCVD system. (b) as-grown GaN-on-Si LED epi-wafer. (c) 10 μm × 10 μm scanning area AFM image of as-grown sample at center region.

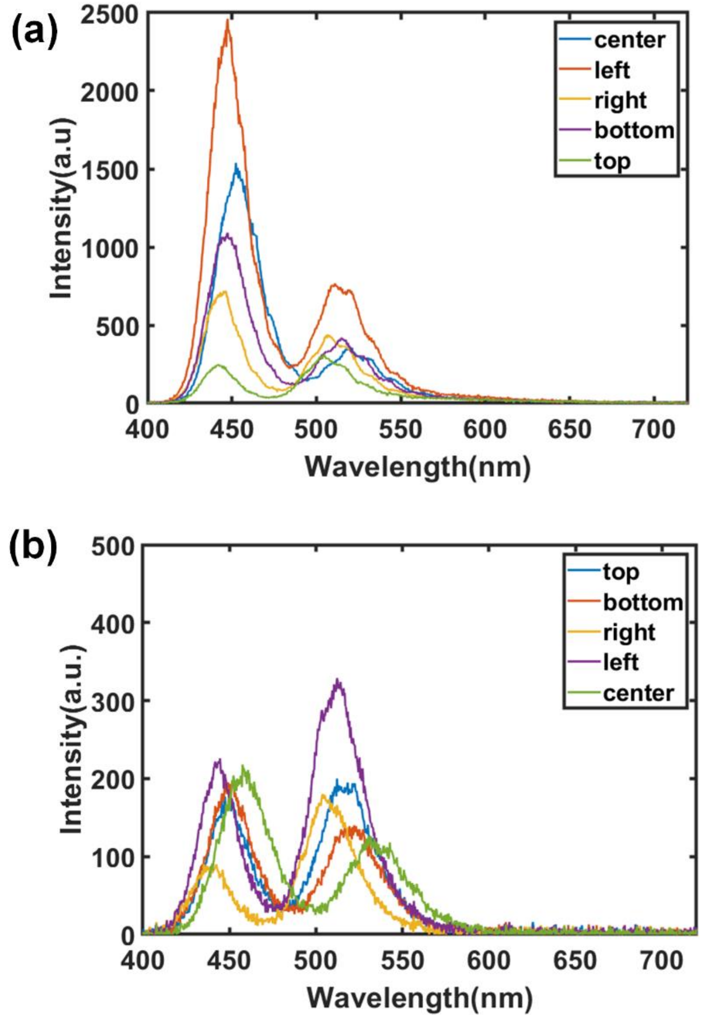

Figure S2. PL and EL characterization. (a) PL spectra (@325nm laser) and (b) quick EL spectra of the 4-inch GaN-on-Si dual-wavelength LED epi-wafer.

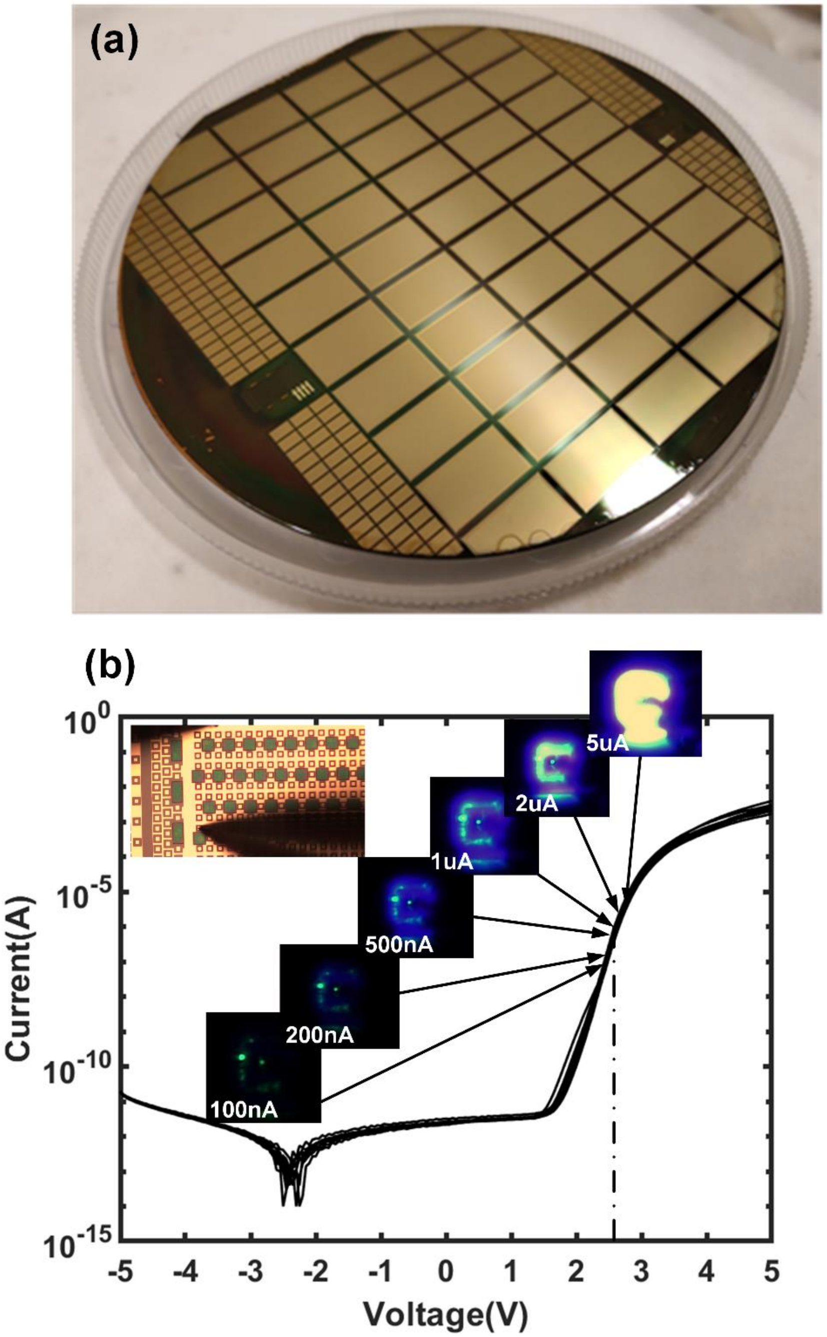

Figure S3. Fabrication and characterization of GaN-on-Si micro-LED arrays. (a) As-fabricated InGaN dual-color micro-LED arrays on 4-inch GaN-on-Si epi-wafer. (b) I-V curves of InGaN dual-color micro-LEDs after metallization (Si substrate was not removal).

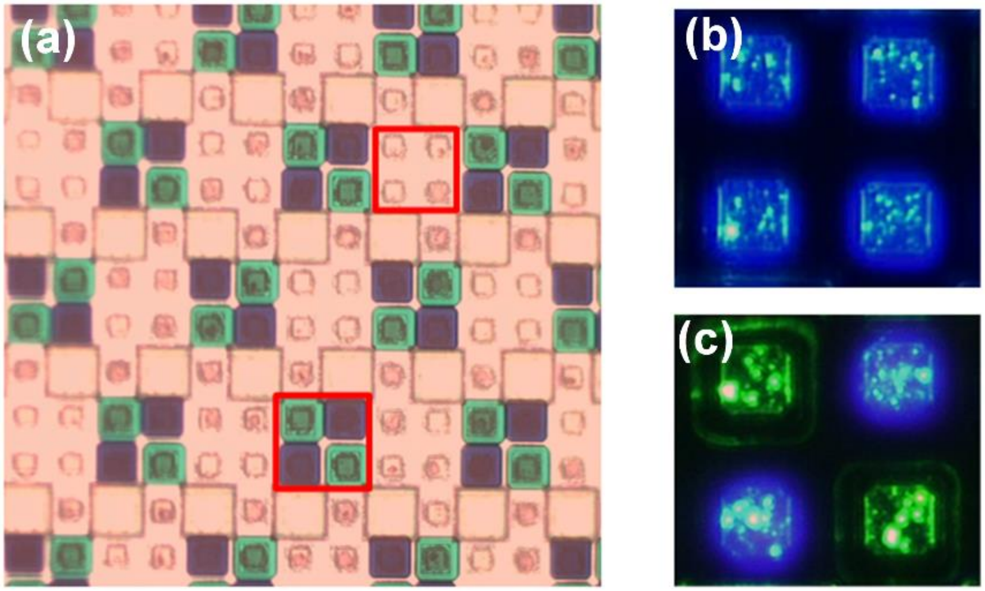

Figure S4. InGaN blue/green dual-color display. (a) After the patterning of blue and green color filters (CFs). (b) Dual-color emission without CFs. (c) Distinguishable blue and green emission with CFs.

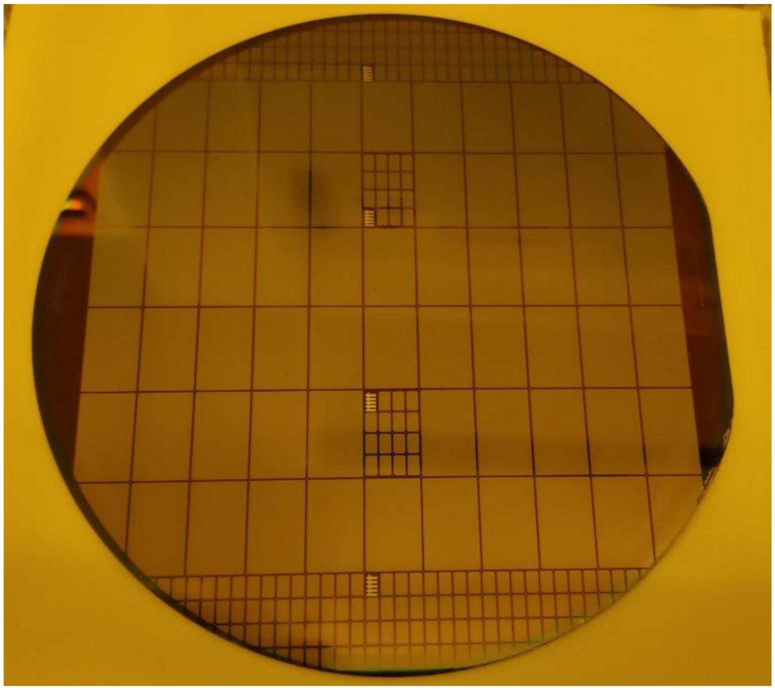

Figure S5. As-fabricated AlGaInP red micro-LED arrays on 4-inch epi-wafer.

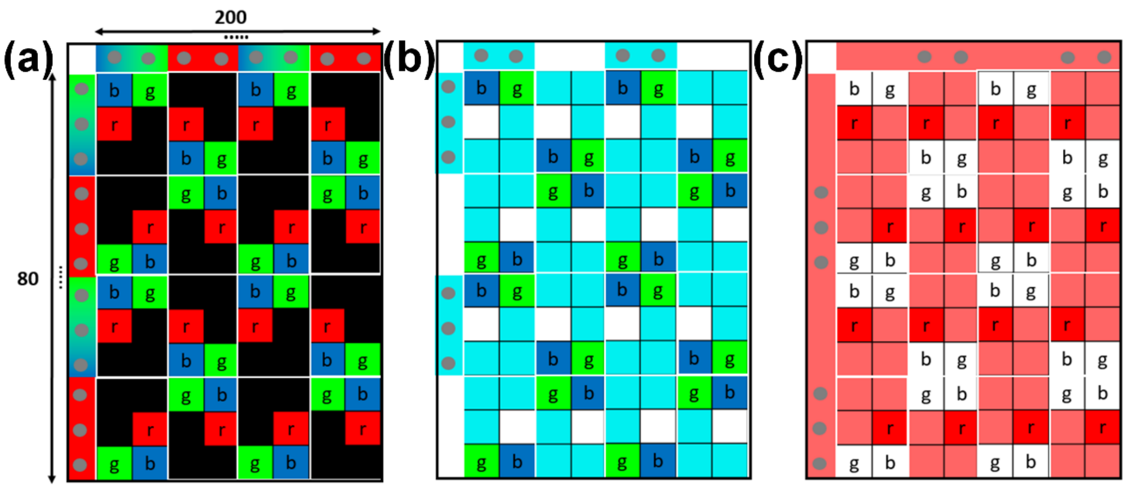

Figure S6. Full-color pixel layout design. (a) Full-color (b) InGaN blue/green dual-color (c) AlGaInP red subpixels configuration with the common-n electrode placed at the peripheral area of the display.

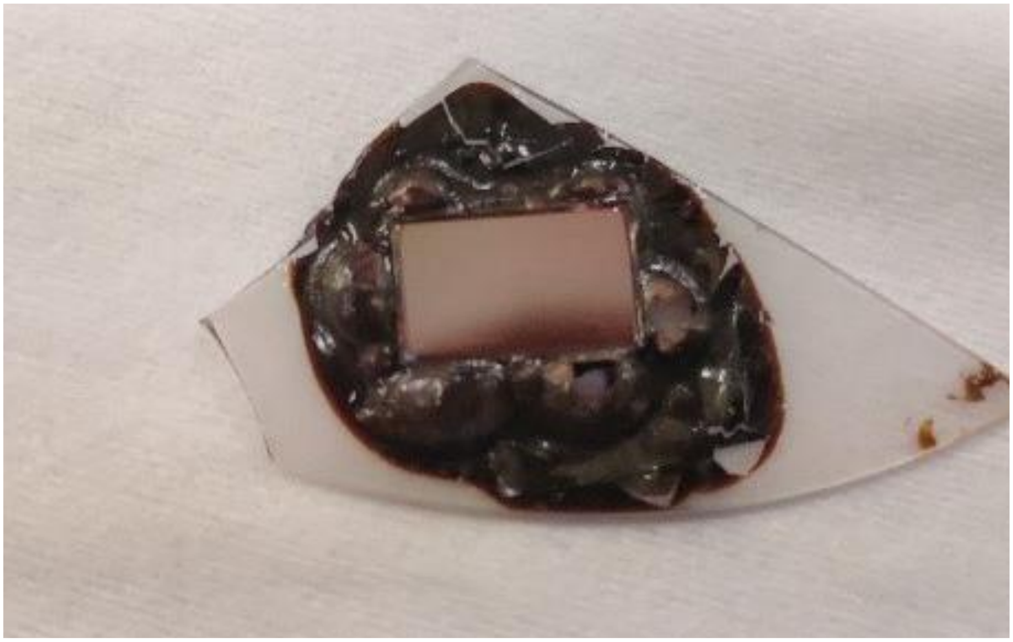

Figure S7. Photograph of the heterogeneous integrated display chip after GaAs substrate removal.

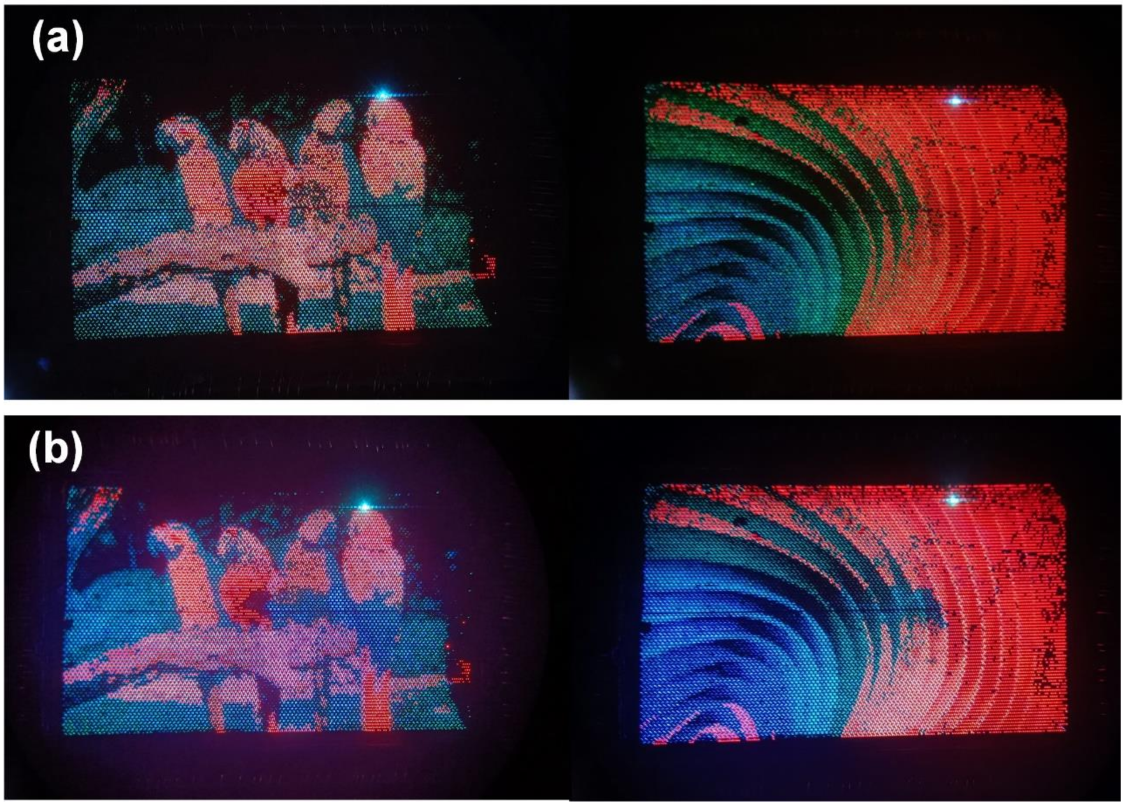

Figure S8. Image demonstration of the full-color display. (a) VLED = 3.2 V. (b) VLED = 5 V.

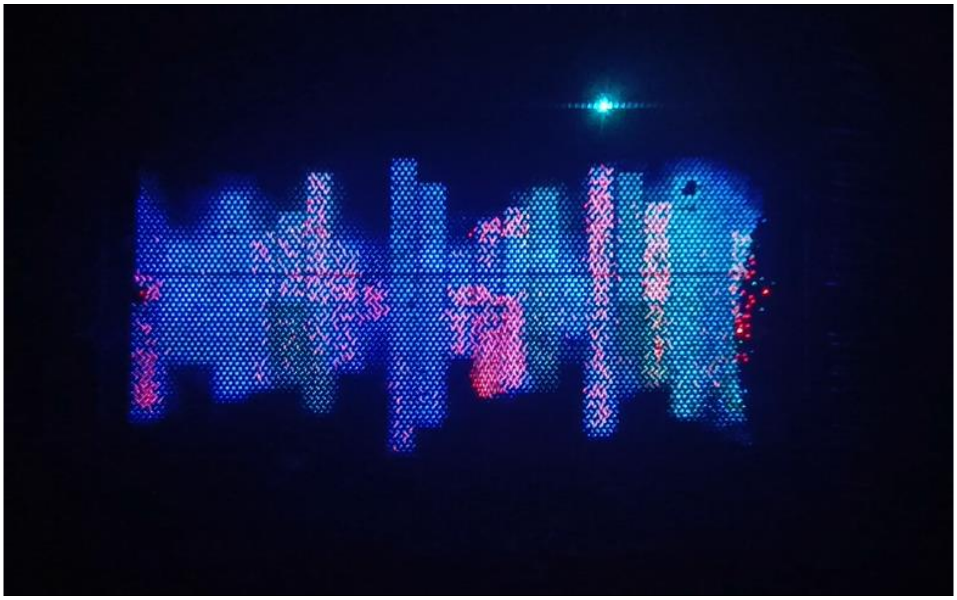

Movie S1. Video demonstration.
